# Prevalence of potentially inappropriate prescribing and prescribing omissions in older Irish adults: findings from **T**he **I**rish **L**ongitu**D**inal Study on **A**geing study (TILDA)

**DOI:** 10.1007/s00228-014-1651-8

**Published:** 2014-02-04

**Authors:** Rose Galvin, Frank Moriarty, Gráinne Cousins, Caitriona Cahir, Nicola Motterlini, Marie Bradley, Carmel M. Hughes, Kathleen Bennett, Susan M. Smith, Tom Fahey, Rose-Anne Kenny

**Affiliations:** 1 HRB Centre for Primary Care Research, Department of General Practice, Royal College of Surgeons in Ireland, 123 St. Stephens Green, Dublin 2, Republic of Ireland; 2 School of Pharmacy, Queen’s University Belfast, Belfast, UK; 3 Department of Pharmacology and Therapeutics, Trinity College Dublin, Dublin, Ireland; 4 The Irish Longitudinal Study of Ageing, Chemistry Extension, Trinity College Dublin, Dublin 2, Ireland; 5 School of Pharmacy, Royal College of Surgeons in Ireland, Dublin, Ireland

**Keywords:** Potentially inappropriate prescribing, Potential prescribing omissions, STOPP, START, Older people

## Abstract

**Purpose:**

We sought to estimate the prevalence of potentially inappropriate prescriptions (PIP) and potential prescribing omissions (PPOs) using a subset of the STOPP/START criteria in a population based sample of Irish adults aged ≥65 years using data from **T**he **I**rish **L**ongitu**D**inal Study on **A**geing (TILDA).

**Methods:**

A subset of 26 PIP indicators and 10 PPO indicators from the STOPP/START criteria were applied to the TILDA dataset. PIP/PPO prevalence according to individual STOPP/START criteria and the overall prevalence of PIP/PPO were estimated. The relationship between PIP and PPOs and polypharmacy, age, gender and multimorbidity was examined using logistic regression.

**Results:**

The overall prevalence of PIP in the study population (*n* = 3,454) was 14.6 %. The most common examples of PIP identified were NSAID with moderate-severe hypertension (200 participants; 5.8 %) and aspirin with no history of coronary, cerebral, or peripheral vascular symptoms or occlusive event (112 participants; 3.2 %). The overall prevalence of PPOs was 30 % (*n* = 1,035). The most frequent PPO was antihypertensive therapy where systolic blood pressure consistently >160 mmHg (*n* = 341, 9.9 %), There was a significant association between PIP and PPO and polypharmacy when adjusting for age, sex and multimorbidity (adjusted OR 2.62, 95 % CI 2.05–3.33 for PIP and adjusted OR 1.46, 95 % CI 1.23–1.75 for prescribing omissions).

**Conclusion:**

Our findings indicate prescribing omissions are twice as prevalent as PIP in the elderly using a subset of the STOPP/START criteria as an explicit process measure of potentially inappropriate prescribing and prescribing omissions. Polypharmacy was independently associated with both PPO and PIP. Application of such screening tools to prescribing decisions may reduce unnecessary medication, related adverse events, healthcare utilisation and cost.

**Electronic supplementary material:**

The online version of this article (doi:10.1007/s00228-014-1651-8) contains supplementary material, which is available to authorized users.

## Introduction

Older adults are a heterogeneous group often presenting with multimorbidity and polypharmacy. Prescribing in older adults is challenging for a number of reasons as any new medication must be considered in the context of the physiological effects of the drug, the absorption and excretion of the drug and age-related changes in body composition and physiology [1]. Potentially inappropriate prescribing (PIP) in older people refers to the use of medications that should be generally avoided in such populations and doses or frequencies of administration that should not be exceeded [2, 3]. Medicines that are considered potentially inappropriate lack evidence based indications, are not cost-effective and may pose a higher risk of adverse events including increases in morbidity, adverse drug events, hospitalisation and mortality [4, 5]. In terms of assessing the appropriateness of prescribing in older people, both implicit and explicit measures of process (providers’ actions) and outcome (e.g., adverse drug events) are used [6]. Implicit process measures involve a clinician’s judgment of appropriateness for the individual patient based on patient characteristics and published work [7]. Explicit process measures are criterion-based and are derived from published evidence based reviews, expert opinion and/or consensus [8, 9].

A comprehensive and explicit process measure of PIP has been developed and validated for use in European countries. The STOPP criteria (Screening Tool for Older Persons’ Prescriptions) are a physiological system based screening tool and comprises 65 clinically significant criteria [10]. The criteria include commonly encountered instances of PIP in older people such as drug–drug and drug–disease interactions, drugs which adversely affect older patients at risk of falls and duplicate drug class prescriptions. In addition, under-prescribing of clinically indicated medications has been identified by an accompanying screening tool known as the START criteria (Screening Tool to Alert doctors to Right Treatment), comprising 22 criteria for potentially appropriate drugs.

There have been few national studies of PIP and PPOs in the general population of older people. In 2007, a subset of the STOPP criteria was applied in a national population study carried out in the Republic of Ireland among people aged ≥70 years, using the Health Services Executive Primary Care Reimbursement Services (HSE-PCRS) pharmacy claims database [6]. This study reported PIP prevalence rates of 36 % in the primary care setting. However, this study was limited in its assessment of PIP as the HSE-PCRS database contains no information on diagnosis and so only a subset of the STOPP criteria could be applied. PIP prevalence rates reported may not have adequately reflected all instances of PIP among older people such as drug-disease interactions. Therefore, the overall aim of the current study is to estimate the prevalence of PIP/PPOs, particularly relating to drug-disease interactions, in a nationally representative sample of individuals aged ≥65 years, using a subset of the STOPP/START criteria.

## Methods

### Study population

The Irish LongituDinal Study on Ageing (TILDA) is a nationally representative cohort study of people aged 50 years and over and resident in Ireland, charting their health, social and economic circumstances over a 10-year period. The first wave of the TILDA study has been completed, and we have examined the data from this cross sectional wave in our current study. The STROBE standardised reporting guidelines for cross-sectional studies have been followed to ensure the uniform conduct and reporting of the research. All participants aged ≥65 years are included [11].

### Overview of TILDA data collection

Data for the current study was captured from three methods used to collect data in the TILDA dataset. Participants first completed a computer-assisted personal interview (CAPI) in their own homes. Detailed information on all aspects of the respondents’ lives was collected in the CAPI, including economic status, health aspects (physical, mental, service needs and usage) and social participation. Participants were then invited to one of two health centres for a comprehensive health assessment. Health assessments were carried out by qualified and trained research nurses. Cognitive, cardiovascular, gait and balance and vision measurements and tests were taken in the health assessment. Finally, each participant was also requested to complete and return a questionnaire designed to explore certain areas that were considered particularly sensitive for respondents to answer directly to an interviewer (e.g., relationship quality, depression and loneliness). The names of medications that participants were taking at the time of the CAPI, including over-the-counter medications, were coded in the TILDA dataset using the World Health Organization (WHO) Anatomical Therapeutic Chemical (ATC) classification system. No information was collected on strength, quantity, duration or cost of prescription.

### Application of the STOPP/START criteria

Inappropriate drug-drug and drug-disease combinations according to the STOPP criteria were identified using information on medications and diagnoses (e.g., hypertension, chronic obstructive pulmonary disease, glaucoma) from the TILDA dataset. In the absence of diagnosis information, prescription drugs for the treatment of certain conditions were used as proxies (e.g., gout, epilepsy). Medications documented by the field-researchers at the CAPI interview were recorded in the TILDA dataset using the WHO ATC classification system. A similar approach was used to identify PPOs. Twenty-six STOPP criteria and ten START criteria were applied to the TILDA database. Table 1 contains details of the criteria applied per physiological system. Reasons for exclusion of criteria are contained in Supplemental Table 1.

**Table 1:** STOPP/START criteria applied to the TILDA dataset

Physiological system	Number of criteria included	Percentage of criteria included
STOPP criteria		
Cardiovascular system	8/17	47.1 %
CNS & psychotropic drugs	4/13	30.8 %
Gastrointestinal system	1/5	20 %
Respiratory system	2/3	66.7 %
Musculoskeletal system	4/8	50 %
Urogenital system	2/6	33.3 %
Endocrine system	1/4	25 %
Drugs that adversely affect those prone to falls	3/5	60 %
Analgesic drugs	0/3	0 %
Duplicate drug classes	1/1	100 %
START criteria		
Cardiovascular system	5/8	62.5 %
Respiratory system	0/3	0 %
Central nervous system	2/2	100 %
Gastrointestinal system	0/2	0 %
Musculoskeletal system	0/3	0 %
Endocrine system	¾	75 %

### Statistical analysis

Data analyses were performed using Stata version 12 (StataCorp, College Station, Tx, USA) and statistical significance at *p* < 0.05 was assumed. Descriptive statistics were used to summarise the population. The overall prevalence of PIP/PPO and the prevalence per individual STOPP/START criteria were calculated as a proportion of all eligible persons ≥65 years. The prevalence of STOPP/START criteria was also calculated as a proportion of the overall disease or drug prevalence (e.g., beta-blocker with COPD as a proportion of COPD prevalence or two concurrent opiates as a proportion of opiate prevalence). Participants were further classified by gender and age group (65–74 years, ≥75 years). Other explanatory variables examined included polypharmacy, defined as concurrent use of five or more medications [12] and number of chronic conditions (none versus 1, 2, 3 or more). Univariate analyses confirmed that explanatory variables (polypharmacy, age, gender and multimorbidity) were significantly associated with PIP/PPOs. The association among any PIP/PPO (versus none) and age, gender, polypharmacy and multimorbidity was assessed using logistic regression and presented as adjusted odds ratios with 95 % confidence intervals.

## Results

### Population descriptive statistics

A total of 3,507 people ≥65 years in Ireland were identified from the TILDA database of which 1,842 (52.5 %) were female and 1,665 (47.5 %) were male. Almost two thirds of individuals were aged between 65 and 74 years (*n* = 2,163). The remaining 38 % (*n* = 1,344) were aged ≥75 years. Fifty-three participants reported missing medication data; theref,ore the STOPP/START criteria were applied to 3,454 participants.

### Overall prevalence of PIP and PPOs

The overall prevalence of PIP was 14.6 % (*n* = 504) considering all 26 STOPP criteria. Almost 13 % of the population (*n* = 433) were prescribed one potentially inappropriate medication, 60 (1.7 %) were prescribed two and 11 (0.3 %) were prescribed three or more. The overall prevalence of PPOs was 30 % (*n* = 1,035) considering the ten included START criteria. Eight hundred and four individuals (23.3 %) presented with one PPO, 188 (5.4 %) with two and 43 (1.2 %) with three or more PPOs.

### Prevalence of PIP according to individual STOPP criteria

Table 2 presents the prevalence of each of the individual STOPP criteria by physiological system. Prescription of a non-steroidal anti-inflammatory drug (NSAID) with moderate-severe hypertension was the most frequent potentially inappropriate drug (*n* = 200, 5.8 %). The second most frequent PIP was aspirin with no history of coronary, cerebral, or peripheral vascular symptoms or occlusive event (*n* = 112, 3.2 %), followed by benzodiazepines in those prone to falls (*n* = 40, 1.2 %) and aspirin with a past history of peptic ulcer disease without a histamine H_2_ receptor antagonist or proton pump inhibitor (*n* = 38, 1.1 %). Angiotensin Converting Enzyme (ACE) inhibitors (*n* = 23, 0.7 %) and NSAIDs (*n* = 21, 0.6 %) were the most frequently prescribed duplicate drugs (Table 2).

**Table 2:** STOPP criteria applied to TILDA data for all those aged ≥65 years in Ireland in 2010

STOPP criteria description	Potentially inappropriate prescription (n)	Potentially inappropriate prescription (%)	Proportionate prescribing per indication (%)^a^
Cardiovascular system			
Loop diuretic as first line-monotherapy for hypertension *(safer, more effective alternatives available)*	9	0.26	0.55
Thiazide diuretic with a history of gout *(may exacerbate gout)*	3	0.09	3.80
Beta-blocker with COPD *(risk of increased bronchospasm)*	31	0.90	18.02
Beta-blocker with verapamil *(risk of symptomatic heart block)*	4	0.12	0.52
Aspirin and warfarin without histamine H_2_ receptor antagonist (except cimetidine) or proton pump inhibitor *(high risk of gastrointestinal bleeding)*	13	0.38	1.00
Dipyridamole as monotherapy for cardiovascular secondary prevention *(no evidence of efficacy)*	5	0.14	0.20
Aspirin with a past history of peptic ulcer disease without histamine H_2_ receptor antagonist or Proton Pump Inhibitor *(risk of bleeding)*	38	1.10	16.38
Aspirin with no history of coronary, cerebral, or peripheral vascular symptoms or occlusive event *(not indicated)*	112	3.24	12.50
Central nervous system and psychotropic drugs			
TCA and glaucoma *(exacerbate glaucoma)*	3	0.09	2.36
TCA and opiate or calcium channel blockers *(risk of severe constipation)*	16	0.46	2.36
Phenothiazines in patients with epilepsy *(may lower seizure threshold)*	3	0.09	2.46
Anticholinergics to treat extra-pyramidal side-effects of neuroleptic medications *(risk of anticholinergic toxicity)*	3	0.09	6.82
Gastrointestinal system			
Prochlorperazine or metoclopramide with parkinsonism *(risk of exacerbating parkinsonism)*	1	0.03	3.23
Respiratory system			
Theophylline as monotherapy for COPD *(safer, more effective alternative: risk of adverse effects due to narrow therapeutic index)*	11	0.32	6.40
Nebulised ipratropium with glaucoma *(exacerbate glaucoma)*	0	–	–
Musculoskeletal system			
NSAID with history of peptic ulcer disease or gastrointestinal bleeding, unless with concurrent histamine H_2_ receptor antagonist, PPI or misoprostol *(risk of peptic ulcer relapse)*	10	0.29	4.31
NSAID with moderate-severe hypertension (moderate: 160/100–179/109 mmHg; severe: ≥180/110 mmHg) *(risk of exacerbation of hypertension)*	200	5.79	10.92
NSAID with heart failure *(risk of exacerbation of heart failure)*	5	0.14	9.26
Warfarin and NSAID *(risk of gastrointestinal bleeding)*	8	0.23	1.57
Urogenital system			
Antimuscarinic drugs with chronic glaucoma (>3 months) *(risk of acute exacerbation of glaucoma)* ^b^	5	0.15	3.94
Alpha-blockers in males with frequent incontinence i.e., one or more episodes of incontinence daily *(risk of urinary frequency and worsening of incontinence)* ^c^	9	0.55	8.18
Endocrine system			
Glibenclamide or chlorpropamide with type 2 diabetes mellitus *(risk of prolonged hypoglycemia)*	3	0.09	0.82
Drugs that adversely affect those prone to falls *(≥ 1 fall in past 3 months)*			
Benzodiazepines *(sedative, may cause reduced sensorium, impair balance)* ^d^	40	1.16	14.55
Neuroleptic drugs *(may cause gait dyspraxia, Parkinsonism)* ^d^	6	0.17	2.18
First generation antihistamines *(sedative, may impair sensorium)* ^d^	1	0.03	0.36
Duplicate drug class prescription *(optimization of monotherapy within a single drug class)*			
Two concurrent opiates	3	0.09	2.26
Two concurrent NSAIDs	21	0.61	5.97
Two concurrent SSRIs	0	–	–
Two concurrent antidepressants	2	0.06	3.03
Two concurrent loop diuretics	0	–	–
Two concurrent ACE inhibitors	23	0.67	2.28

*COPD* chronic obstructive pulmonary disease, *TCA* tricyclic antidepressant, *NSAID* non-steroidal anti-inflammatory drug, *SSRI* selective serotonin reuptake inhibitor, *ACE inhibitors* angiotensin converting enzyme inhibitors and Angiotensin receptor blockers

^a^Proportionate prescribing per indication, e.g., prevalence of STOPP criteria as a proportion of the overall disease or drug prevalence, e.g.,Beta-blocker with COPD as a proportion of COPD prevalence, two concurrent opiates as a proportion of opiates prevalence

^b^8 (0.23 %) missing data for chronic glaucoma variable

^c^Proportion of male participants only, 5 (0.30 %) missing data for urinary incontinence variable

^d^1 (0.03 %) missing data for falls in past year variable

### Prevalence of PPOs according to individual START criteria

Table 3 presents the prevalence of each of the individual START criteria by physiological system. The most frequent PPO was antihypertensive therapy where systolic blood pressure consistently >160 mmHg (*n* = 341, 9.9 %), followed by the omission of warfarin in the presence of chronic atrial fibrillation (*n* = 270, 7.8 %) and statin therapy in diabetes mellitus if one or more co-existing major cardiovascular risk factors were present (*n* = 235, 6.8 %).

**Table 3:** START (Screening Tool to Alert doctors to Right Treatment) criteria applied to TILDA data for all those aged ≥65 years in Ireland in 2010

START criteria description	Potential prescribing omissions (n)	Potential prescribing omissions (%)	Proportionate prescribing omission per indication (%)*
Cardiovascular system			
Warfarin in the presence of chronic atrial fibrillation	270	7.82	75.00
Antihypertensive therapy where systolic blood pressure consistently >160 mmHg †	341	9.87	18.62
Angiotensin Converting Enzyme (ACE) inhibitor with chronic heart failure	23	0.67	42.59
ACE inhibitor following acute myocardial infarction	126	3.65	47.19
Beta-blocker with chronic stable angina	151	4.37	45.21
Central nervous system			
L-DOPA in idiopathic Parkinson’s disease with definite functional impairment and resultant disability	3	0.09	17.65
Antidepressant drug in the presence of moderate-severe depressive symptoms lasting at least 3 months §	44	1.30	70.97
Endocrine system			
ACE inhibitor or Angiotensin Receptor Blocker in diabetes with nephropathy, i.e., overt urinalysis proteinuria	13	0.38	44.83
Antiplatelet therapy in diabetes mellitus if one or more co-existing major cardiovascular risk factor present (hypertension, hypercholesterolaemia, smoking history)	110	3.18	35.48
Statin therapy in diabetes mellitus if one or more co-existing major cardiovascular risk factor present	235	6.80	75.81

*Proportionate prescribing omission per indication, e.g. prevalence of PPO as a proportion of the overall disease, e.g. no warfarin with chronic atrial fibrillation as a proportion of chronic atrial fibrillation prevalence

^§^70 (2.03 %) missing data for depressive symptoms variable

^†^1,119 (32.40 %) missing data for blood pressure variable

### Factors associated with PIP

Any PIP was more likely in people ≥75 years than those 65–74 years when adjusting for gender (OR 1.41, 95 % CI 1.16–1.70). This association remained significant after additionally adjusting for polypharmacy (age ≥75 years vs. 65–74 years, OR 1.22, 95 % CI 1.00–1.50). There was no association between PIP and gender when adjusting for age (F vs. M, OR 1.05, 95 % CI 0.87–1.26). Supplemental Table 2 presents the association between gender and age and PIP by individual STOPP criteria. There was a strong association between any PIP and polypharmacy when accounting for age and gender (OR 3.13, 95 % CI 2.53–3.87), and this association remained significant when additionally adjusting for the presence of chronic conditions (OR 2.62, 95 % CI 2.05–3.33).

### Factors associated with PPOs

There was no difference in the likelihood of a PPO in those ≥75 years when compared to those 65–74 years when adjusting for gender (OR 1.09, 95 % CI 0.92–1.28). However, omissions were significantly more likely in males when compared to females when adjusting for age (OR 0.73, 95 % CI 0.63–0.85). The association between PPOs and gender remained significant after also adjusting for polypharmacy (OR 0.71, 95 % CI 0.61–0.83). Supplemental Table 3 presents the association between gender and age and PPOs by individual START criteria. There was a significant association between PPOs and polypharmacy when adjusting for age and gender (OR 2.28, 95 % CI 1.93–2.69) and this association also remained significant when also adjusting for number of chronic conditions (OR 1.46, 95 % CI 1.23–1.75).

## Discussion

### Statement of principal findings

This current study found that almost 15 % of people aged ≥65 years in the Republic of Ireland received at least one PIP, according to a subset of 26 STOPP criteria. PIP was more likely in older adults (≥75 years) when adjusting for gender and polypharmacy. The overall prevalence of potential prescribing omissions was 30 % considering the ten included START criteria. Omissions were significantly more likely in males when compared to females when adjusting for age and polypharmacy. There was a significant association between PIP/PPOs and polypharmacy when adjusting for age, gender and multimorbidity.

### Results in the context of current literature

This study presents a snapshot of the prevalence of PIP in a nationally representative population of people aged ≥65 years using data from the first wave of TILDA. Previous research is limited by having focused on specific groups in particular clinical settings as well as having measured PIP using Beers’ criteria derived in the US [13–16]. A limited number of studies of PIP in the general population of older people exist. In a previous Irish study using data from the HSE-PCRS, an overall prevalence of 36 % in patients ≥70 years was reported using a subset of 30 STOPP criteria [6]. Similar results were reported in a study of the Enhanced Prescribing Database (EPD) in Northern Ireland where the overall prevalence of PIP was 34 % [5]. However, the differences between the TILDA database and these prescription reimbursement databases make comparisons more difficult. The lack of clinical information relating to diagnoses (e.g., hypertension, CVA) in the EPD and PRCS databases and the lack of information on drug duration, dose or frequency of administration of prescriptions in the TILDA dataset mean that only a limited number of the STOPP criteria applied in the three studies are similar. In the EPD and PCRS databases, the most prevalent PIP drugs were proton pump inhibitors (PPIs) at maximum therapeutic dosage for >8 weeks, followed by NSAIDs for >3 months and long-acting benzodiazepines for >1 month [5, 6]. These instances of PIP were not captured in TILDA. Similarly, the use of a NSAID with moderate-severe hypertension was the most prevalent PIP in TILDA, and this could not be assessed in the prescription databases due to a lack of clinical information. Of the 19 STOPP criteria that were the same in PCRS and TILDA, the prevalence of PIPs are broadly similar, but low in both datasets (<2.5 %).

The significant association between PIP and polypharmacy is consistent with findings reported in previous studies [5, 6, 17], even when age, gender and multimorbidity is accounted for [18]. While it is well recognised that the use of multiple medications in the older adults is associated with increased risk of adverse events such as adverse drug events in patients taking NSAIDs (e.g., gastrointestinal haemorrhage) and increased incidence of falls and cognitive impairment in those taking sedative-hypnotic drugs, these medications are routinely among the most common PIPs [3, 6, 19], as was the case in our study. We also found an association between advancing age and PIP after adjusting for polypharmacy, similar to a previous primary care based study [20]. We reported no association between PIP and gender after adjustments for age. These findings are contrary to previous studies reporting that women are more likely to receive a PIP when compared with men [6, 17, 21].

Our overall findings relating to the prevalence of PPOs are similar to previous studies that have used the START criteria in a primary care setting [20]. Other studies have reported higher prevalence rates of omissions among an older hospital inpatient population [22, 23]. However, older patients admitted to hospital are sicker and frailer than those in the community so the findings need to be considered in the context of the population of interest. The cardiovascular system accounted for the majority of PPOs in keeping with previous reports [20]. Our study demonstrated that polypharmacy is associated with the under-prescribing of indicated medicines even when adjusting for age, gender and co-morbid conditions. Similar findings have been reported in other studies, particularly those with co-morbid conditions [24, 25]. However, these findings warrant further investigation.

We also noted that over 70 % of study participants who reported moderate-severe depressive symptoms were not prescribed an antidepressant drug. Depressive symptoms were assessed in TILDA using the Center for Epidemiologic Studies Depression Scale (CES-D) [26]. We used a cut off score of ≥27 points on the CES-D scale as a proxy for diagnosis of ‘moderate-severe’ depressive symptoms. This interpretation is the most conservative estimate reported in the literature [27]; therefore, our findings need to be considered in the context of these short-comings. It may be that other management approaches were adopted in the treatment of depressive symptoms such as cognitive behavioural therapy [28].

### Clinical and policy implications

The term ‘potentially’ is used as there is often limited evidence to support the inclusion of particular medications in lists of drugs to avoid in particular populations. However, their use or lack thereof may be indicated and appropriate in certain circumstances considering the clinical presentation of the patient and the balance of the risk and benefits [5]. Clinical vigilance and quality efforts should place particular focus on medication appropriateness, both with respect to under-prescribing and over-prescribing. Reducing PIP in older people will require implementation of more robust methods of medication reviews to routinely assess drug effectiveness, dosage, duration, interactions and adverse effects [29, 30].

The updated NICE guidelines (2013) on falls assessment and prevention in older people highlight the importance of a medication review in older people at risk of falls who present to healthcare professionals. There is a lack of experimental research on the use of the STOPP criteria as a tool to reduce the incidence of adverse drug events (ADEs) such as falls in older people. However, a recent randomised controlled trial demonstrated that the application of the STOPP criteria significantly improved medication appropriateness when compared with the usual pharmaceutical care in 400 hospital inpatients [3]. The study was not powered to detect a reduction in secondary outcomes such as ADEs but further research is warranted to examine the role of medicines reviews based on the STOPP criteria as an intervention tool to attenuate ADE incidence. In addition, reducing the number of drugs used by older people may serve to reduce the risk of falls and the associated direct and indirect costs [29].

### Strengths and weaknesses of the study

The TILDA database contains data on morbidities (>99.5 % complete) that enable the assessment of PIP in the context of a given diagnosis (e.g., hypertension, cardiovascular conditions). These data also facilitate the assessment and measurement of errors of omission, i.e., the lack of use of appropriate drugs when needed. However, it was only possible to apply a subset of the STOPP/START criteria to the database, as described in Table 1, due to a lack of information on drug strength, dose and duration of prescriptions. This may have led to an underestimate of the true prevalence of PIP/PPO among older people in TILDA. In addition, the validity of the original criteria is diminished by only applying a subset and the comparability to other population based studies is limited due to the differences in criteria applied.

The cost of PIP was not considered in the present study, but a previous study estimated the cost of PIP to be in excess of €45 million using a subset of 30 STOPP criteria [6]. Further research is planned to link the TILDA dataset to the national prescribing database (HSE-PCRS) to facilitate the application of 19 additional STOPP criteria and to estimate the cost of PIP using these linked datasets. In addition, there is a need to compare these findings with other European population based studies. The association between explicit process measures of PIP and prescribing omissions, and health outcomes (morbidity and mortality) and healthcare utilisation also requires further investigation to facilitate the development and testing of appropriate interventions to reduce PIP and PPOs in older adults. In the Irish context, this can be completed using future waves of the TILDA data.

## Conclusion

Our findings indicate PIP and PPOs are prevalent in the elderly using a subset of the STOPP/START criteria as an explicit process measure of potentially inappropriate prescribing and PPOs. Application of such screening tools to prescribing decisions may reduce unnecessary medication, related adverse events, healthcare utilisation and cost.

## Electronic supplementary material

Below is the link to the electronic supplementary material.

ESM 1 (DOC 28 kb)

ESM 2 (DOC 59 kb)

ESM 3 (DOC 35 kb)
