# Supplementary material for: Prevalence of potentially inappropriate prescribing and prescribing omissions in older Irish adults: findings from The Irish LongituDinal Study on Ageing study (TILDA)
Source: Eur J Clin Pharmacol. 2014 Feb 4;70(5):599–606. doi: 10.1007/s00228-014-1651-8 (PMC3978378; doi:10.1007/s00228-014-1651-8)
Supplement: Supplementary file 1 — (DOC 28 kb) [file 228_2014_1651_MOESM1_ESM.doc]

**Supplemental Table 1:** Reasons for excluding STOPP/START criteria

| ***STOPP criteria*** |
| --- |
| Thirty-nine STOPP criteria were excluded as the variables required to apply the criteria were not collected of the as part of the TILDA study. More specifically, STOPP criteria were excluded the for the following reasons: the disease/condition and/or severity was not recorded and no drug proxy was suitable (e.g. oedema, constipation, renal failure) (n=20), the duration of prescription was not collected (n=14), no information on drug dosage was collected (n=2) and individuals with dementia were excluded from participating in the TILDA study - this precluded three further criteria. |
| ***START criteria*** |
| Twelve START criteria were excluded for the following reasons: the disease/condition and/or severity was not collected as part of the TILDA study and no drug proxy was suitable (e.g. gastro-oesophageal reflux, constipation, renal impairment) (n=9) and the duration of prescription not collected (n=3). |
