# Supplementary material for: Prevalence of potentially inappropriate prescribing and prescribing omissions in older Irish adults: findings from The Irish LongituDinal Study on Ageing study (TILDA)
Source: Eur J Clin Pharmacol. 2014 Feb 4;70(5):599–606. doi: 10.1007/s00228-014-1651-8 (PMC3978378; doi:10.1007/s00228-014-1651-8)
Supplement: Supplementary file 2 — (DOC 59 kb) [file 228_2014_1651_MOESM2_ESM.doc]

**Supplemental Table 2:** The association between gender and age and PIP by individual STOPP criteria in 2010

| **STOPP Criteria Description** | **OR gender with 95% CI***  **(F vs. M)** | | | **OR age with 95% CI***  **(≥75 vs. 65-74 years)** | |
| --- | --- | --- | --- | --- | --- |
| ***Cardiovascular System*** | |  |  | |  |
| Loop diuretic as first line-monotherapy for hypertension *(safer, more effective alternatives available)* | | 3.36 (0.66-17.06) | 3.18 (0.72-13.94) | |  |
| Thiazide diuretic with a history of gout *(may exacerbate gout)* | | - | 0.59 (0.05-6.52) | |  |
| Beta-blocker with COPD *(risk of increased bronchospasm)* | | 0.67 (0.31-1.44) | 0.69 (0.30-1.59) | |  |
| Beta-blocker with verapamil *(risk of symptomatic heart block)* | | 1.58 (0.17-14.41) | 0.98 (0.14-6.90) | |  |
| Aspirin and warfarin without histamine H_2_ receptor antagonist (except cimetidine) or proton pump inhibitor  *(high risk of gastrointestinal bleeding)* | | 0.11 (0.01-0.88) | 1.25 (0.41-3.89) | |  |
| Dipyridamole as monotherapy for cardiovascular secondary prevention *(no evidence of efficacy)* | | 0.16 (0.02-1.52) | 4.35 (0.47-40.60) | |  |
| Aspirin with a past history of peptic ulcer disease without histamine H2 receptor antagonist or Proton Pump Inhibitor *(risk of bleeding)* | | 0.54 (0.26-1.10) | 0.71 (0.34-1.49) | |  |
| Aspirin with no history of coronary, cerebral, or peripheral vascular symptoms or occlusive event *(not indicated)* | | 0.94 (0.64-1.40) | 2.20 (1.49-3.24) | |  |
| ***Central Nervous System and psychotropic drugs*** | |  |  | |  |
| TCA and glaucoma *(exacerbate glaucoma)* | | 1.44 (0.12-17.39) | - | |  |
| TCA and opiate or calcium channel blockers *(risk of severe constipation)* | | 2.88 (0.78-10.66) | 1.40 (0.51-3.87) | |  |
| Phenothiazines in patients with epilepsy *(may lower seizure threshold)* | | 1.44 (0.15-13.62) | 1.74 (0.18-16.45) | |  |
| Anticholinergics to treat extra-pyramidal side-effects of neuroleptic medications *(risk of anticholinergic toxicity)* | | 1.85 (0.16-21.59) | - | |  |
| ***Gastrointestinal System*** | |  |  | |  |
| Prochlorperazine or metoclopramide with parkinsonism *(risk of exacerbating parkinsonism)* | | - | - | |  |
| ***Respiratory System*** | |  |  | |  |
| Theophylline as monotherapy for COPD *(safer, more effective alternative: risk of adverse effects due to narrow therapeutic index)* | | 0.72 (0.22-2.31) | 0.58 (0.16-2.12) | |  |
| Nebulised ipratropium with glaucoma *(exacerbate glaucoma)* | | - | - | |  |
| ***Musculoskeletal System*** | |  |  | |  |
| NSAID with history of peptic ulcer disease or gastrointestinal bleeding, unless with concurrent histamine H_2_ receptor antagonist, PPI or misoprostol *(risk of peptic ulcer relapse)* | | 0.93 (0.26-3.34) | 0.63 (0.14-2.75) | |  |
| NSAID with moderate-severe hypertension (moderate: 160/100mmHg – 179/109mmHg; severe: ≥180/110mmHg) *(risk of exacerbation of hypertension)* | | 1.62 (1.19-2.20) | 0.84 (0.61-1.16) | |  |
| NSAID with heart failure *(risk of exacerbation of heart failure)* | | 1.29 (0.21-7.90) | 0.68 (0.11-4.36) | |  |
| Warfarin and NSAID *(risk of gastrointestinal bleeding)* | | 1.02 (0.24-4.35) | 8.36 (0.98-71.27) | |  |
| ***Urogenital System*** | |  |  | |  |
| Antimuscarinic drugs with chronic glaucoma (> 3 months) *(risk of acute exacerbation of glaucoma)* § | | 1.38 (0.22-8.61) | 2.98 (0.44-20.27) | |  |
| Alpha-blockers in males with frequent incontinence i.e. one or more episodes of incontinence daily *(risk of urinary frequency and worsening of incontinence)* € | | - | 2.76 (1.00-22.04) | |  |
| ***Endocrine System*** | |  |  | |  |
| Glibenclamide or chlorpropamide with type 2 diabetes mellitus *(risk of prolonged hypoglycemia)* | | 0.62 (0.05-7.48) | - | |  |
| **Drugs that adversely affect those prone to falls *(≥ 1 fall in past three months)*** | |  |  | |  |
| Benzodiazepines *(sedative, may cause reduced sensorium, impair balance)* ¥ | | 1.50 (0.78-2.89) | 1.51 (0.76-3.00) | |  |
| Neuroleptic drugs *(may cause gait dyspraxia, Parkinsonism)* ¥ | | 0.92 (0.19-4.39) | 3.05 (0.65-14.43) | |  |
| First generation antihistamines *(sedative, may impair sensorium)* ¥ | | - | - | |  |
| **Duplicate Drug Class Prescription *(optimization of monotherapy within a single drug class)*** | |  |  | |  |
| Two concurrent opiates | | 0.38 (0.03-5.16) | 1.90 (0.14-25.80) | |  |
| Two concurrent NSAIDs | | 3.39 (1.14-10.12) | 1.44 (0.54-3.82) | |  |
| Two concurrent SSRIs | | - | - | |  |
| Two concurrent antidepressants | | 0.39 (0.02-7.81) | 1.85 (0.09-37.01) | |  |
| Two concurrent loop diuretics | | - | - | |  |
| Two concurrent ACE inhibitors | | 0.76 (0.33-1.74) | 2.26 (0.88-5.81) | |  |

*OR Gender= odds ratio adjusted for age and polypharmacy.

*OR Age= odds ratio adjusted for gender and polypharmacy.

COPD= chronic obstructive pulmonary disease, TCA= tricyclic antidepressant, NSAID= non-steroidal anti-inflammatory drug, SSRI= selective serotonin reuptake inhibitor, ACE inhibitors= Angiotensin converting enzyme inhibitors and Angiotensin receptor blockers.

§ 8 (0.23%) missing data for chronic glaucoma variable.

€ proportion of male participants only, 5 (0.30%) missing data for urinary incontinence variable.

¥ 1 (0.03%) missing data for falls in past year variable

- = unable to calculate
