# Supplementary material for: Prevalence of potentially inappropriate prescribing and prescribing omissions in older Irish adults: findings from The Irish LongituDinal Study on Ageing study (TILDA)
Source: Eur J Clin Pharmacol. 2014 Feb 4;70(5):599–606. doi: 10.1007/s00228-014-1651-8 (PMC3978378; doi:10.1007/s00228-014-1651-8)
Supplement: Supplementary file 3 — (DOC 35 kb) [file 228_2014_1651_MOESM3_ESM.doc]

**Supplemental Table 3:** The association between gender and age and prescribing omissions by individual START criteria in 2010

| **START Criteria Description** | **OR gender with 95% CI***  **(F vs. M)** | | **OR age with 95% CI***  **(≥75 vs. 65-74 years)** | |
| --- | --- | --- | --- | --- |
| ***Cardiovascular System*** |  | | |  |
| Warfarin in the presence of chronic atrial fibrillation | 0.82 (0.63-1.08) | | | 1.17 (0.88-1.54) |
| Antihypertensive therapy where systolic blood pressure consistently >160 mmHg † | 1.02 (0.82-1.28) | | | 0.81 (0.63-1.04) |
| Angiotensin Converting Enzyme (ACE) inhibitor with chronic heart failure | 0.39 (0.15-0.98) | | | 0.84 (0.33-2.12) |
| ACE inhibitor following acute myocardial infarction | 0.33 (0.21-0.50) | | | 1.49 (1.03-2.16) |
| Beta-blocker with chronic stable angina | 0.86 (0.61-1.22) | | | 1.52 (1.08-2.15) |
| ***Central Nervous System*** |  | | |  |
| L-DOPA in idiopathic Parkinson’s disease with definite functional impairment and resultant disability | | - | | 7.43 (0.68-81.19) |
| Antidepressant drug in the presence of moderate-severe depressive symptoms lasting at least three months § | | 1.73 (0.93-3.19) | | 0.52 (0.26-1.05) |
| ***Endocrine System*** | |  | |  |
| ACE inhibitor or Angiotensin Receptor Blocker in diabetes with nephropathy i.e. overt urinalysis proteinuria | | 1.32 (0.43-4.02) | | 1.11 (0.37-3.28) |
| Antiplatelet therapy in diabetes mellitus if one or more co-existing major cardiovascular risk factor present (hypertension, hypercholesterolaemia, smoking history). | | 0.84 (0.57-1.25) | | 1.01 (0.65-1.56) |
| Statin therapy in diabetes mellitus if one or more co-existing major cardiovascular risk factor present. | | 0.65 (0.49-0.87) | | 0.83 (0.61-1.13) |

*OR Gender= odds ratio adjusted for age and polypharmacy.

*OR Age= odds ratio adjusted for gender and polypharmacy.

§ 70 (2.03%) missing data for depressive symptoms variable.

- = unable to calculate
